# Personal and familial factors associated with toilet training

**DOI:** 10.1590/S1677-5538.IBJU.2020.0129

**Published:** 2020-11-18

**Authors:** Jose Murillo B., Juliane Cristine de Paula, Cassandra Ribeiro Bastos, Daniela Gonçalves Soares, Nathália Cristina Toledo de Castro, Katia Kalianne do Vale Sousa, Ademar Vasconcellos do Carmo, Ricelly Lignani de Miranda, Flávia Cristina de Carvalho Mrad, José de Bessa

**Affiliations:** 1 Faculdade de Ciências Médicas e da Saúde de Juiz de Fora Juiz de ForaMG Brasil Faculdade de Ciências Médicas e da Saúde de Juiz de Fora – SUPREMA, Juiz de Fora, MG, Brasil; 2 Universidade Federal de Juiz de Fora Faculdade de Medicina Juiz de ForaMG Brasil Faculdade de Medicina da Universidade Federal de Juiz de Fora – UFJF, Juiz de Fora, MG, Brasil; 3 Universidade Federal de Minas Gerais Faculdade de Medicina Unidade de Nefrologia Pediátrica Belo HorizonteMG Brasil Unidade de Nefrologia Pediátrica, Faculdade de Medicina de Universidade Federal de Minas Gerais - UFMG, Belo Horizonte, MG, Brasil; 4 Universidade Estadual de Feira de Santana Faculdade de Medicina Feira de SantanaBA Brasil Faculdade de Medicina de Universidade Estadual de Feira de Santana – UEFS, Feira de Santana, BA, Brasil

**Keywords:** Toilet Training, Personal Health Services, Family

## Abstract

**Purpose:**

Toilet training (TT) is an important marker in a child's physical and psychosocial development. The present study aimed to evaluate aspects associated to delayed TT.

**Material and Methods:**

We interviewed 372 parents of children who had completed TT up to 48 months before the interview. The questionnaires were applied at school exits when parents went to pick their children up and at public parks. Questions included demographics, aspects related to TT, dysfunction voiding symptom score and evaluation of constipation.

**Results:**

The interviews were performed at a mean of 15.3±10.4 (0 to 47) months after the end of TT. Girls accounted for 53% of the sample. The mean age at finishing TT was 31.6±9.3 months and similar in both genders (p=0.77). TT occurred before school entry in 45.7% of the children and medical advice for TT was sought only by 4.8% of the parents. No association was observed of age at completing TT and presence of lower urinary tract symptoms (LUTS) (p=0.57) and/or constipation (p=0.98). In the univariate analysis, prematurity (OR=2.7 [95% CI 2.3-3.1], p <0.0001) and mothers who work outside their household (OR=1.8 [95% CI 1.4-2.3], p <0.0001) were associated to delayed TT.

**Conclusion:**

Children completed TT at a mean of 2 years and 7 months of age. The age of completing TT was not related to LUTS and/or constipation. Premature children and those whose mothers work outside the home finish TT later.

## INTRODUCTION

Toilet training (TT) is defined as the ability to independently start and complete micturition and defecation. It is an important marker in a child's normal development, being a challenging process not only for the children but also for their parents. In the past decades an increase in the age of toilet training has been observed (1–3). The American Academy of Pediatrics recommends a Child-Oriented Approach for TT, in which should only be started when the child is physically and psychologically ready (1, 2).

Data on acquisition of bladder and bowel control is conflicting with studies showing no differences between genders, and others demonstrating an earlier control in girls (4–6). Acquiring bladder control, in many occasions, occurs at the same time as bowel control. Accomplishing nighttime urinary continence may also occur at the same time as daytime continence or be several months delayed (4–8).

Many different factors have been associated with the age of TT, such as age of the mother, parental education level (9), working status of the mother, socioeconomic status of the family (10), and single parenthood, race (4, 11), gender with girls being trained earlier than boys (4, 8) and developmental delays (5). On the other hand, other studies have not found associations between TT and working status of the mother (4, 8), socioeconomic status of the family (8), prematurity (4, 5, 8) nor with mild to moderate neurological impairment (8).

The literature on TT is scarce and shows many controversies. We hypothesized that child development and familial environment may influence in the age a child completes TT. Therefore, the aim of the present study is to evaluate personal and household factors associated with toilet training as well as if there is any correlation between bladder and bowel dysfunction and the age TT is completed.

## MATERIALS AND METHODS

A questionnaire was applied to parents of children who had completed TT no more than 48 months before the interview. Parents were interviewed in public areas, such as parks or in front of schools and the interview lasted about 10 minutes. Children whose parents refused to participate, presenting any urogenital disorder, who were using any medications or presented with any disease known to interfere on bladder or bowel function, severe intellectual or motor disability, and those who had not finished TT were not included in the study. Age at which toilet training was completed was defined as the age that the child had full bladder and bowel control, without any failure in holding urine or stool during day and nighttime.

The institutional review board on ethics approved the study (protocol number 1.757.676) and all parents signed an informed consent.

The questionnaires included: 1) demographic questions on gender, socioeconomic status, neonatal history, marital status of the parents, if both parents worked full time or not; 2) aspects related to TT (Appendix-1); 3) current voiding and bowel symptoms assessed by the dysfunctional voiding symptom score (DVSS) (12), Pediatric Rome III Criteria (13) and Modified Bristol Stoll Form Scale for Children (14).

### Statistical Analysis

Data were expressed as means±SD, medians and interquartile ranges, or absolute values and fractions. The Student t or Mann-Whitney U test was used to compare continuous variables while categorical variables were compared using the chi-square or Fisher's exact test.

Analysis of risk factors for delayed toilet training was performed by multivariable binary logistic regression with stepwise forward likelihood ratio method. Odds ratios (OR) were reported with a 95% confidence interval. Tests of significance were two-tailed, and significance was defined by p <0.05.

All tests were 2-sided with p <0.05 considered statistically significant and were performed using MedCalc Statistical Software version 18.11.6.

## RESULTS

Of the 400 questionnaires applied, 372 were included in the study. Of the 28 not included, 15 were due to refusal of parents or caregivers to participate in the study, and the remaining did not fulfill inclusion criteria. The mean time between TT and the interview was 15.3±10.4 (0 a 47) months. Girls represented 53% of the sample. All other demographic data are described on Table-1.

**Table 1:** Demographic Data.

			Frequency	%
**Pre or After Pre-school**				
	TT prior starting Pre-school		170	45.7
		TT after starting Pre-school	202	54.3
**Total**			372	100
**What made parents start TT**				
		Orientation of a Physician	18	4.8
		School Orientation	75	20.3
		Parents thought it was time to begin	162	43.5
		Children gave signs that he/she was ready	95	25.5
		Others	22	5.9%
**Total**			372	100
**Problems during TT**				
		None	329	88.4
		Refuse to go to the bathroom	17	4.7
		Retention Maneuvers	16	4.3
		Hiding or Asking for diapers to poop	5	1.3
		Others	5	1.3
**Total**			372	100
TT Methods**				
		Brazelton (child-oriented approach)	346	93
		Azrin and Foxx (parent-oriented approach)	2	0.5
		Alarm	1	0.3
		Others	23	6.2
**Total**			372	100
Equipment Used for TT***				
		Potty Chair	225	43.6
		Regular Toilet	149	28.9
		Toilet with support for feet	45	8.7
		Toilet Seat Reducer	94	18.2
		Other	3	0.6
**Total**			516	100
**Parent's view of the ideal age to start TT**				
		< 12 months	6	1.6
		12 to 18 months	42	11.3
		18 to 24 months	95	25.5
		24 to 30 months	149	40.1
		30 to 36 months	62	16.7
		> 30 months	18	4.8
**Tota**l			372	100

* TT Toilet Training

** Methods adapted from Choby et al., (1) and Brazelton et al., (7).

*** Some parents use more than one equipment.

The mean age at the moment of the interview was 46.5 months (female: 46.7±12.0 and male: 46.3±11.4 months; p=0.77) and the mean age for TT was 31.6±9.3 months (median: 30.0 months), being 31.1±9.4 for girls and 32.2±9.2 for boys (p=0.20). No differences were found between TT for bowel (day and night) and bladder (day and night) according to gender or type of TT (Table-2).

**Table 2:** Time to TT (bowel and bladder) and gender.

Gender and Age at TT				
	All Children	Boys	Girls	
TT	Mean age (months) (min-max)	Mean age (min-max)	Mean age (min-max)	p value
Bowel daytime	29.1 ± 8.6 (12-60)	30.0 ± 8.6 (12-60)	28.4 ± 8.5 (12-56)	0.07
Bowel nighttime	29.3 ± 8.7 (11-60)	30.0 ± 8.8 (12-60)	28.7 ± 8.5 (11-52)	0.14
Bladder daytime	28.4 ± 8.1 (9-56)	28.9 ± 7.4 (12-48)	28.0 ± 8.6 (9-56)	0.31
Bladder nighttime	30.7 ± 9.1 (9-62)	30.9 ± 8.7 (12-60)	30.6 ± 9.5 (9-62)	0.72

**TT** = Toilet training; *p* value < 0.05.

We used the 75-percentile value that was 36 months to divide the sample for binary further analysis.

Evaluating the family, age of the mother (p=0.10) and father (p=0.66), schooling of the householder (p=0.27), socioeconomic class (p=0.47) had no impact on the age of completing TT. The only familial factor associated with a delayed TT was mothers that work outside of their household (p <0.001) (Table-3).

**Table 3:** Familial aspects related to toilet training (TT).

Schooling and Age of TT						
				Mother		Father
Schooling		Age at TT		Number (%)		Number (%)
Elementary School		< 36 months		70 (23.8)		67 (25.0)
		> 36 months		15 (20.5)		12 (17.1)
High School		< 36 months		113 (38.3)		105 (39.2)
		> 36 months		27 (37.0)		22 (32.4)
College		< 36 months		69 (23.4)		50 (18.7)
		> 36 months		16 (21.9)		21 (30.9)
Post-Graduation		< 36 months		43 (14.6)		46 (17.2)
		> 36 months		15 (20.5)		13 (19.1)
**Total**				**386**		**336**
p value		Pearson Chi-Square		0.37		0.21
**Working Status of Parents and Age of TT**						
**Working Status**		**Age at TT**		**Mother Number (%)**		**Father Number (%)**
Yes		< 36 months		201 (67.9)		263 (96.0)
		> 36 months		58 (79.5)		67 (97.1)
No		< 36 months		95 (32.1)		11 (4.0)
		> 36 months		15 (20.5)		2 (2.9)
**Total**				**369**		**343**
p value		Pearson Chi-Square		< 0.001		0.66
**Age of Parents and Age of TT**						
**Age of Parents (Years)**		**Age at TT**		**Mother Number (%)**		**Father Number (%)**
< 20		< 36 months		34 (11.4)		12 (4.0)
		> 36 months		4 (5.4)		2 (2.7)
20 – 30		< 36 months		151 (50.7)		137 (46.0)
		> 36 months		47 (63.5)		38 (51.4)
> 30		< 36 months		113 (37.9)		149 (50.0)
		> 36 months		23 (31.1)		34 (45.9)
**Total**				**372**		**372**
p value		Pearson Chi-Square		0.10		0.66
**Socioeconomic Class and Age of TT**						
**Age at TT**	**A Number (%)**		**B Number (%)**	**C Number (%)**	**D/E Number (%)**	**Total Number (%)**
< 36 months	29 (9.9)		139 (47.8)	107 (36.8)	16 (5.5)	291 (100)
> 36 months	12 (14.4)		36 (49.3)	23 (31.5)	2 (2.7)	73 (100)
**Total**	**41 (11.3)**		**175 (48.1)**	**130 (35.7)**	**18 (4.9)**	**364 (100)**
**P value: 0.47**						

Analyzing the association between age of TT and bladder and bowel dysfunction (BBD) it was observed that age at the end of TT had no impact on the development of LUTS (p=0.56) nor functional constipation (p=0.89 and p=0.45, respectively) (Table-4).

**Table 4:** Lower Urinary Tract Dysfunction, Constipation and Age of Toilet Training (TT).

Dysfunctional Voiding Symptoms Score (DVSS)				
Age at TT	No LUTD Number (%)		LUTD Number (%)	Total Number (%)
< 36 months	267 (89.6)		31 (10.4)	298 (100)
> 36 months	68 (91.9)		6 (8.1)	74 (100)
Total	335 (90.1)		37 (9.9)	372 (100)
		**P=0.56**		
**Constipation (Modifed Bristol Stool Scale for Children)**				
**Age at TT**	**No Constipation Number (%)**		**Constipation Number (%)**	**Total Number (%)**
< 36 months	157 (54.3)		132 (45.7)	289 (100)
> 36 months	35 (47.9)		38 (52.1)	73 (100)
Total	192 (53.0)		170 (47.0)	362 (100)
		**p=0.45**		
**Constipation (Rome III)**				
**Age at TT**	**No Constipation Number (%)**		**Constipation Number (%)**	**Total Number (%)**
< 36 months	243 (81.5)		55 (18.5)	298 (100)
> 36 months	59 (80.8)		14 (19.2)	73 (100)
**Total**	**302 (81.4)**		**69 (18.6)**	**371 (100)**
		**p=0.89**		

In a univariate analysis, only prematurity (OR=2.7 [95% CI 2.3-3.1], p <0.0001) and mothers who work outside household (OR=1.8 [95% CI 1.42.3], p <0.0001) were associated with a delayed TT. The same factors remained as independent predictors in the multivariate analysis (OR=3.9 [95% CI 3.0-5.1] and OR=1.6 [95% CI 1.2-2.1], p <0.0001, respectively).

## DISCUSSION

Toilet Training is an important step in a child's development, and it depends on child's growth and maturation. As in many other developmental delays seen on a preterm born child (15), we have also demonstrated that these children become toilet trained at a later age. Prematurity has been shown to be associated with motor, cognitive, academic, language and behavior problems (15, 16). Therefore, a delayed TT would be expected in these children since it is a personal achievement related to behavioral and developmental maturation. According to our findings, being a premature child is an independent factor, with 2.7 times greater chance of completing TT after 3 years of age when compared to full term children.

Voluntary control of micturition is mediated by the cerebral cortex that is responsible for facilitating and inhibiting the micturition reflex. Therefore, the ability to gain bladder control is a complex developmental process that can be influenced by anatomical, physiological, cultural, and behavioral conditions (1, 17). Contradictory to our finding, some previous reports have shown that age of completing TT is not associated to prematurity (5, 8, 11, 18). On the other hand, two studies done 20 years apart have shown a delay in starting TT (>24 months) in preterm compared to full term children but both failed to find this difference in the age of completing it (8, 18).

Since our data were collected interviewing parents up to 48 months after their children completed TT, we did not inquire about the age of starting training due to a considerable memory bias.

Despite the fact that most studies show no association between these two variables, working status of the parents, especially of the mother, is currently identified as the major cause of a delayed TT (10, 11). In opposition to these studies, our findings support the hypothesis that mothers that work outside their household, is an independent factor associated with 1.8 greater chance of their children presenting a delayed TT. Parents play an essential role in training their children to get control of their elimination functions providing orientation and guiding the whole training process. If the parents are not at home or are too busy with working and housekeeping there will be a lack of attention to their children TT process which, ultimately, will lead to its delay.

Besides the working status of the mother, the use of disposable diapers and the availability of more information on the Child-Oriented Approach for TT have also been reported as related to this increasing age for completing TT (2, 19). Therefore, parents were asked about different TT methods such Brazelton Child-Oriented Approach that suggests starting the toilet training process only when children show readiness signs; Azrin and Foxx Parent-Oriented Approach that represents an intensive structured behavioral method (“toilet training in a day”); Daytime Wetting Alarm, Child-Assisted Toilet Training that begins bowel and bladder training at two to three weeks of age and the child is taken to the bathroom after a large meal or when showing signs of elimination and the Elimination Communication that guides start of TT at birth and shows how to recognize children's body language, noise and elimination patterns (1, 7), and were also asked about equipment used on TT. As to our study, the majority of families (93%) used the child-oriented approach to toilet train their children, indicating that this could really be a reason for a later TT, as the mean age for completing TT in our group of children was 31.6 months, similar to that reported in other studies (4, 5, 7, 10). When assessing the type of equipment used during TT, 43% of the children used a potty chair and 28.9% used the regular toilet without seat reducer and/or feet support. It is important to note that the incorrect posture while urinating and evacuating can lead to retention maneuvers and BBD (20, 21). Therefore, children should always use a toilet with seat reducer and feet support, or a potty chair in order to determine a sense of security and a more physiological position to facilitate urination and evacuation.

There is a widespread believe that girls end TT at an earlier age. Shum et al. studying the sequential of acquisition of TT skills described that normal developed girls achieve nearly all skills prior to boys (4) and few other studies have confirmed these findings (8, 11), which are in disagreement with ours, as well as of other authors finding (6, 7, 10), where no differences were found between genders regarding age of completing TT. It is also believed by many that bowel control occurs prior to bladder control. A study from Stein & Susser in the sixties described a sequence of on acquirement of sphincter control with bowel control occurring before daytime control and daytime bladder control before nighttime control (22) which was also confirmed by Schum et al. (4) On the opposite direction, we and others have not found differences in time of acquisition of bladder and bowel control (6) adding to the controversies on many of the aspects related to TT.

One of the greatest debates related to TT is whether an earlier or later TT is associated to BBD, although few studies have investigated these associations. As data present herein, other studies found no relationship between age or method use for TT (1, 2, 23) with the development of LUTS. However, Barone et al. reported a higher prevalence of urge-incontinence in children who started later their TT (3). In another recent study, voiding dysfunction was associated with TT starting either before 24 months or after 36 months of age in the presence of constipation (24).

Evaluating the presence of functional constipation, we also found that age of acquiring bladder control is not related to constipation, which is in agreement with the findings of Wald et al. (25). On the contrary, Inan et al. demonstrated a threefold risk for constipation in children that acquire bowel control after the age of 2 years (26). Aziz et al. have also suggested that later TT would predispose to constipation, although their study compared different populations, from rural and urban areas, with other confounding factors (27). Most of the data on bowel function reported in the literature is focused in stool withholding, hiding to defecate, and toilet refusal. Children who hides to defecate and failure to acquire TT for stool elimination at 42 months of age have a delay in bladder control and those who have problems during TT are more prone to present toilet refusal, stool withholding, functional constipation and primary encopresis (28).

There are some limitations to this study. Although we tried to evaluate children who had finished their TT no later than 48 months, since a memory bias could have influenced the results, for this matter we may have missed long-term problems, such as BBD, related to TT. We also failed to evaluate age at the beginning and time taken to complete TT.

## CONCLUSION

Children completed TT at two and a half years of age. Both girls and boys completed TT at a similar age. Premature children and those whose mothers work outside their household are at increased risk of completing TT at a later age. We could not demonstrate association between age at completing TT to LUTS or functional constipation..

## Appendix 1 Questionnaire toilet training for parents or caregivers.

**Appendix 1:** Questionnaire toilet training for parents or caregivers

**1 - Neonatal History**	
( ) Born at full Term ( ) Premature (≤ 37 weeks). Gestational Age: _______ weeks ( ) He was admitted to a neonatal ICU. Why? ______________	
**2 - Parent's working status**	
	2.1 Mother works outside household ( ) No ( ) Yes (part time) ( ) Yes (full time) 2.2 Father works outside household ( ) No ( ) Yes (part time) ( ) Yes (full time)
**3 - Who does the child live with?**	
	( ) parentes ( ) mother ( ) father ( ) Other family member. Who?_____________
**4 - What method did you use to toilet train your child?**	
( ) Child-Oriented Approach (the child gave signs that it was time to start) (Brazelton) ( ) You thought it was the right time to remove the diapers ( ) Removed the diapers in one day (Azin and Foxx) ( ) Taught your son/daughter from birth and he/she has never worn diapers ( ) Used alarm system ( ) Other. Which?_____________________________	
**5. What type of equipment did you use during toilet training?**	
( ) Potty chair ( ) Regular toilet ( ) Toilet with footrest ( ) Toilet with seat reducer ( ) Toilet with seat reducer and footrest ( ) Other. Which?_____________________________	
**6. At what age your child finished toilet training?**	
( ) Did not retire the diapers Age at which acquired bladder control during the day_____months Age at which acquired bladder control during the night_____months Age at which acquired bowel control during the day_____months Age at which acquired bowel control during the night_____months	

## References

[1] 1. Choby BA, George S. Toilet training. Am Fam Physician. 2008;78:1059-64. 19007052

[2] 2. Colaco M, Johnson K, Schneider D, Barone J. Toilet training method is not related to dysfunctional voiding. Clin Pediatr (Phila). 2013;52:49-53. 10.1177/0009922812464042 23117239

[3] 3. Barone JG, Jasutkar N, Schneider D. Later toilet training is associated with urge incontinence in children. J Pediatr Urol. 2009;5:458-61. 10.1016/j.jpurol.2009.05.012 19570720

[4] 4. Schum TR, Kolb TM, McAuliffe TL, Simms MD, Underhill RL, Lewis M. Sequential acquisition of toilet-training skills: a descriptive study of gender and age differences in normal children. Pediatrics. 2002;109:E48. 10.1542/peds.109.3.e48 11875176

[5] 5. Mota DM, Barros AJ. Toilet training: methods, parental expectations and associated dysfunctions. J Pediatr (Rio J). 2008;84:9-17. 10.2223/JPED.1752 18264618

[6] 6. Largo RH, Molinari L, von Siebenthal K, Wolfensberger U. Does a profound change in toilet-training affect development of bowel and bladder control? Dev Med Child Neurol. 1996;38:1106-16. 10.1111/j.1469-8749.1996.tb15074.x 8973296

[7] 7. Brazelton TB, Christophersen ER, Frauman AC, Gorski PA, Poole JM, Stadtler AC, et al. Instruction, timeliness, and medical influences affecting toilet training. Pediatrics. 1999;103(6 Pt 2):1353-8. 10353953

[8] 8. Largo RH, Molinari L, von Siebenthal K, Wolfensberger U. Development of bladder and bowel control: significance of prematurity, perinatal risk factors, psychomotor development and gender. Eur J Pediatr. 1999;158:115-22. 10.1007/s004310051030 10048607

[9] 9. Joinson C, Heron J, Von Gontard A, Butler U, Emond A, Golding J. A prospective study of age at initiation of toilet training and subsequent daytime bladder control in school-age children. J Dev Behav Pediatr. 2009;30:385-93. 10.1097/dbp.0b013e3181ba0e77 19827219

[10] 10. Koc I, Camurdan AD, Beyazova U, Ilhan MN, Sahin F. Toilet training in Turkey: the factors that affect timing and duration in different sociocultural groups. Child Care Health Dev. 2008;34:475-81. 10.1111/j.1365-2214.2008.00829.x 18485025

[11] 11. Schum TR, McAuliffe TL, Simms MD, Walter JA, Lewis M, Pupp R. Factors associated with toilet training in the 1990s. Ambul Pediatr. 2001;1:79-86. 10.1367/1539-4409(2001)001<0079:fawtti>2.0.co;2 11888377

[12] 12. Calado AA, Araujo EM, Barroso U Jr, Netto JM, Filho MZ, Macedo A Jr, et al. Cross-cultural adaptation of the dysfunctional voiding score symptom (DVSS) questionnaire for Brazilian children. Int Braz J Urol. 2010;36:458-63. 10.1590/s1677-55382010000400009 20815952

[13] 13. Rasquin A, Di Lorenzo C, Forbes D, Guiraldes E, Hyams JS, Staiano A, et al. Childhood functional gastrointestinal disorders: child/adolescent. Gastroenterology. 2006;130:1527-37. 10.1053/j.gastro.2005.08.063 PMC7104693 16678566

[14] 14. Lane MM, Czyzewski DI, Chumpitazi BP, Shulman RJ. Reliability and validity of a modified Bristol Stool Form Scale for children. J Pediatr. 2011;159:437-441.e1. 10.1016/j.jpeds.2011.03.002 PMC3741451 21489557

[15] 15. Synnes A, Hicks M. Neurodevelopmental Outcomes of Preterm Children at School Age and Beyond. Clin Perinatol. 2018;45:393-408. 10.1016/j.clp.2018.05.002 30144845

[16] 16. Blencowe H, Cousens S, Chou D, Oestergaard M, Say L, Moller AB, et al. Born Too Soon Preterm Birth Action Group. Born too soon: the global epidemiology of 15 million preterm births. Reprod Health. 2013;10(Suppl 1):S2. 10.1186/1742-4755-10-S1-S2 PMC3828585 24625129

[17] 17. Griffiths D. Neural control of micturition in humans: a working model. Nat Rev Urol. 2015;12:695-705. 10.1038/nrurol.2015.266 26620610

[18] 18. Yildiz D, Suluhan D, Eren Fidanci B, Mert M, Tunc T, Altunkaynak B. The Differences Between Preterm and Term Birth Affecting Initiation and Completion of Toilet Training Among Children: A Retrospective Case-Control Study. Urol J. 2019;16:180-5. 10.22037/uj.v0i0.4820 31004337

[19] 19. van Nunen K, Kaerts N, Wyndaele JJ, Vermandel A, Hal GV. Parents’ views on toilet training (TT): A quantitative study to identify the beliefs and attitudes of parents concerning TT. J Child Health Care. 2015;19:265-74. 10.1177/1367493513508232 24270991

[20] 20. Braga AANM, Veiga MLT, Ferreira MGCDS, Santana HM, Barroso U Jr. Association between stress and lower urinary tract symptoms in children and adolescents. Int Braz J Urol. 2019;45:1167-79. 10.1590/S1677-5538.IBJU.2019.0128 PMC6909859 31808405

[21] 21. Altunkol A, Abat D, Sener NC, Gulum M, Ciftci H, Savas M, et al. Is urotherapy alone as effective as a combination of urotherapy and biofeedback in children with dysfunctional voiding? Int Braz J Urol. 2018;44:987-95. 10.1590/S1677-5538.IBJU.2018.0194 PMC6237517 30130020

[22] 22. Stein Z, Susser M. Social factors in the development of sphincter control. Dev Med Child Neurol. 1967;9:692-706. 10.1111/j.1469-8749.1967.tb02352.x 6081889

[23] 23. Vasconcelos MMA, East P, Blanco E, Lukacz ES, Caballero G, Lozoff B, et al. Early Behavioral Risks of Childhood and Adolescent Daytime Urinary Incontinence and Nocturnal Enuresis. J Dev Behav Pediatr. 2017;38:736-42. 10.1097/DBP.0000000000000516 PMC5679404 29045258

[24] 24. Hodges SJ, Richards KA, Gorbachinsky I, Krane LS. The association of age of toilet training and dysfunctional voiding. Res Rep Urol. 2014;6:127-30. 10.2147/RRU.S66839 PMC4199658 25328866

[25] 25. Wald ER, Di Lorenzo C, Cipriani L, Colborn DK, Burgers R, Wald A. Bowel habits and toilet training in a diverse population of children. J Pediatr Gastroenterol Nutr. 2009;48:294-8. 10.1097/mpg.0b013e31817efbf7 19274784

[26] 26. Inan M, Aydiner CY, Tokuc B, Aksu B, Ayvaz S, Ayhan S, et al. Factors associated with childhood constipation. J Paediatr Child Health. 2007;43:700-6. 10.1111/j.1440-1754.2007.01165.x 17640287

[27] 27. Aziz S, Moiz Fakih HA, Di Lorenzo C. Bowel habits and toilet training in rural and urban dwelling children in a developing country. J Pediatr. 2011;158:784-8. 10.1016/j.jpeds.2010.11.024 21183187

[28] 28. Taubman B, Blum NJ, Nemeth N. Children who hide while defecating before they have completed toilet training: a prospective study. Arch Pediatr Adolesc Med. 2003;157:1190-2. 10.1001/archpedi.157.12.1190 14662572

